# Significance of tumor mutation burden and immune infiltration in thymic epithelial tumors

**DOI:** 10.1111/1759-7714.14002

**Published:** 2021-05-25

**Authors:** Zi‐Ming Wang, Qi‐Rong Xu, David Kaul, Mahmoud Ismail, Harun Badakhshi

**Affiliations:** ^1^ Charité ‐ Universitätsmedizin Berlin, Corporate Member of Freie Universität Berlin, Humboldt‐Universität zu Berlin, and Berlin Institute of Health Berlin Germany; ^2^ Department of Thoracic Surgery, Klinikum Ernst von Bergmann Academic Hospital of Charité ‐ Universitätsmedizin Berlin Potsdam Germany; ^3^ School of Medicine Zhengzhou University Zhengzhou China; ^4^ Department of Radiation Oncology Charité‐Universitätsmedizin Humboldt University Berlin Berlin Germany; ^5^ Department of Radiation Oncology, Klinikum Ernst von Bergmann Academic Hospital of Charité ‐ Universitätsmedizin Berlin Potsdam Germany

**Keywords:** immune infiltration, prognosis, thymic epithelial tumors, thymus, tumor mutation burden

## Abstract

**Background:**

Thymic epithelial tumors (TETs) are relatively rare malignant thoracic tumors. Tumor mutation burden (TMB) and immune infiltration play important roles in tumorigenesis.

**Methods:**

Research data was obtained using the Cancer Genome Atlas (TCGA) database to evaluate the landscape of tumor mutations, related factors, and relationship of prognosis. The CIBERSORT algorithm was used to evaluate immune cell infiltration in TETs and its relationship with TMB. Immune‐related differentially expressed genes (irDEGs) were identified. Hub irDEGs independently related to prognosis were analyzed using univariate and multivariate Cox proportional hazard models. A survival signature was constructed from hub irDEGs.

**Results:**

A total of 122 patients were included in this study. GTF2I was the most common gene mutation. Higher TMB was significantly associated with the later stage, more advanced pathological type, and older age. The overall survival (OS) of patients in the low‐TMB group was significantly better. There was no significant correlation between TMB levels and PD‐L1 expression. Enrichment analysis showed that DEGs were mainly involved in the P13K–Akt signaling pathway. There were significant differences in macrophage and other types of immune cell infiltration between the high‐ and low‐TMB groups. CCR5, FASLG, and CD79A independently relating to prognosis were screened from 391 irDEGs. The low‐risk group had a significantly better prognosis than the high‐risk group based on the signature, which has a good predictive effect on OS.

**Conclusions:**

In this study, TETs patients with high TMB had a significantly poor prognosis and an immune‐related gene signature was found to effectively evaluate the long‐term prognosis.

## Graphical abstract

Thymic epithelial tumors (TETs) patients with high tumor mutation burden (TMB) had a significantly poor prognosis. There was a significantly higher TMB in older patients, later stages, and thymic carcinoma. The study also revealed that there was no significant association between TMB and PD‐L1 expression.

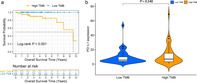

## INTRODUCTION

Thymic epithelial tumors (TETs) are primary neoplasms that originate from thymic epithelial cells or differentiate into the thymic epithelium. TETs are the most common anterior mediastinal tumors, with an annual incidence of 0.15/100000, and include thymoma and thymic carcinoma (TC).^1^ TETs are mainly treated with surgery, radiotherapy, chemotherapy, and other multidisciplinary treatments. Due to their relatively low incidence, the molecular biological characteristics of TETs remain unclear.^2^ The analysis of the pathogenesis and molecular biological characteristics of TETs has clinical significance for further development of treatment strategies.

Tumor mutation burden (TMB) refers to the total number of somatic gene nonsynonymous mutations per million bases in a particular region of the tumor genome. TMB, which can be used to predict the effect of immunotherapy, has been an independent predictive biomarker for tumor immunotherapy.^3^ In recent years, an increasing number of studies have shown that tumors with higher nonsynonymous mutation burden tend to form more new antigens, and the higher the TMB of the tumor, the more likely a patient is to benefit from immune checkpoint inhibitor therapy.^4^ However, at present, there are relatively few studies on TMB in TETs.

With the rapid development of sequencing technology and the deepening of basic research on the molecular pathways and molecular mechanisms of tumorigenesis, precise targeting therapy based on gene mutations has become a new phenomenon in the prevention and treatment of many kinds of tumors.^5^ In this study, we used bioinformatic data to analyze the TMB correlation with TETs in the basic mechanism of gene mutation, to analyze the tumor biological characteristics and prognosis of patients with different TMB, and to analyze the difference between immune infiltration and tumor microenvironment.

## METHODS

### Data acquisition

The main data used in this study included tumor mutation and transcriptome profiles, clinical information, and immune‐related data. With the support of the GDC data portal (https://portal.gdc.cancer.gov/), we collected TETs somatic mutation data from the Cancer Genome Atlas (TCGA) database THYM dataset, which is publicly available. Data file subtypes, masked somatic mutation, processed based on the VarScan software, were selected for further analysis. Moreover, transcriptome profiles in which all available TETs samples had an HTSeq‐FPKM workflow were downloaded. The GDC portal was used to collect clinical data, such as sex, age, race, pathological type, prior malignancy, Masaoka‐Koga (MK) stage, myasthenia gravis history, overall survival (OS) time, and survival outcomes. The ImmPort database was used to identify the immune‐related genes. No ethical conflict was declared, given all the study data from public databases.

### Evaluation of tumor mutation burden

According to the definition of TMB, Perl scripts were used to calculate the mutation frequency on the JAVA8 platform, where exon length/variant number (38 million) for each sample was considered. The whole exon somatic mutation data in the TCGA database were in the mutation annotation format (MAF). The “maftools” R package offering multiple analysis modules was implemented to calculate the TMB data according to the amount of coding errors for each sample, the proportion of gene variation classification, the proportion of single nucleotide variation, the proportion of mutant genes, and perform the visualization process. The TETs samples were classified according to the median value into low‐ and high‐TMB groups. The TMB value was merged with the corresponding clinical data, including survival information, via the ID number of each sample. The associations of TMB levels with clinical characteristics between different groups of variables were measured using the Wilcoxon rank‐sum test. The expression of programmed death‐ligand 1 (PD‐L1; official symbol name: CD274) was evaluated in all samples to calculate the differential expression of this gene between high and low TMB groups and visualized using the violin plot. The survival curves of the low‐ and high‐TMB groups were plotted using the Kaplan–Meier method, and the log‐rank test was used to evaluate the significance of the difference in prognosis between the two groups.

### Screening of differentially expressed genes and analysis of functional pathways

Normalization was applied to the transcriptome profiles of each patient with TETs. Differentially expressed genes (DEGs) were examined with the “limma” R package in the two TMB groups, where false discovery rate (FDR) <0.05, and fold change (FC) >1 were adopted. The significant DEGs between the two groups are shown on a heatmap with the “pheatmap” R package. The Kyoto Encyclopedia of Genes and Genomes (KEGG) and Gene Ontology (GO) enrichment analysis were performed on DEGs with the “clusterProfiler” R package. A dot plot on the “ggplot2” R package was used to show the functional pathway analysis results.

### Analysis of immune cell infiltration and immune‐related gene identification

The CIBERSORT algorithm is a deconvolution tool that evaluates the gene expression matrix of 22 human leukocyte subtypes using linear support vector regression. The relative percentage of infiltrating immune cells was calculated using this tool from the transcriptional data of each TET sample. The differential abundances of immune cell infiltration between the two groups were compared and analyzed using the Wilcoxon ranked‐sum test. The gene sets of immune‐related genes obtained from the ImmPort database (https://www.immport.org/shared/genelists) (Updated: July 2020) and DEGs were combined, and the intersection genes were identified as immune‐related DEGs (irDEGs). A Venn plot was used to show the relationship between the two sets.

### Protein‐to‐protein interaction (PPI) analysis and prognosis survival analysis of hub irDEGs

The STRING database was used to build a PPI network of the irDEGs. The interaction network of irDEGs was visualized using the Cytoscape software. The upregulated genes are shown in red, while the downregulated genes are shown in blue. The number of network clusters for each immune‐related DEG was calculated to identify the hot interaction genes. A univariate Cox proportional hazard model prognosis analysis was performed for each irDEG. Those genes with *p* < 0.02, were included in multivariate Cox analysis. A prognostic immune signature for OS was constructed from irDEGs that were independently related to prognosis. The median signature score was used to divide patients into high‐and low‐risk groups. The log‐rank test and Kaplan–Meier curve were used to analyze their differential survival outcomes. The predictive value of the immune gene signature in TETs was evaluated using the receiver operating characteristic curve (ROC).

### Statistical analysis

The Kaplan–Meier “survival” package was applied to the Cox regression model. The log‐rank test and Kaplan Meier method were used to formulate survival curves. A chi‐square (X2) test was used to compare categorical variables. Differential analysis and normalization were adopted on the basis of the “Limma” package. As a nonparametric statistical hypothesis test, the Wilcoxon rank‐sum test was applied to the comparative analysis between the two groups. The R project (Version 4.0.3) laid the basis for all statistical analyses, where significance was identified by *p* < 0.05.

## RESULTS

### Summary of tumor mutation profiles in thymic epithelial tumors

**FIGURE 1:**
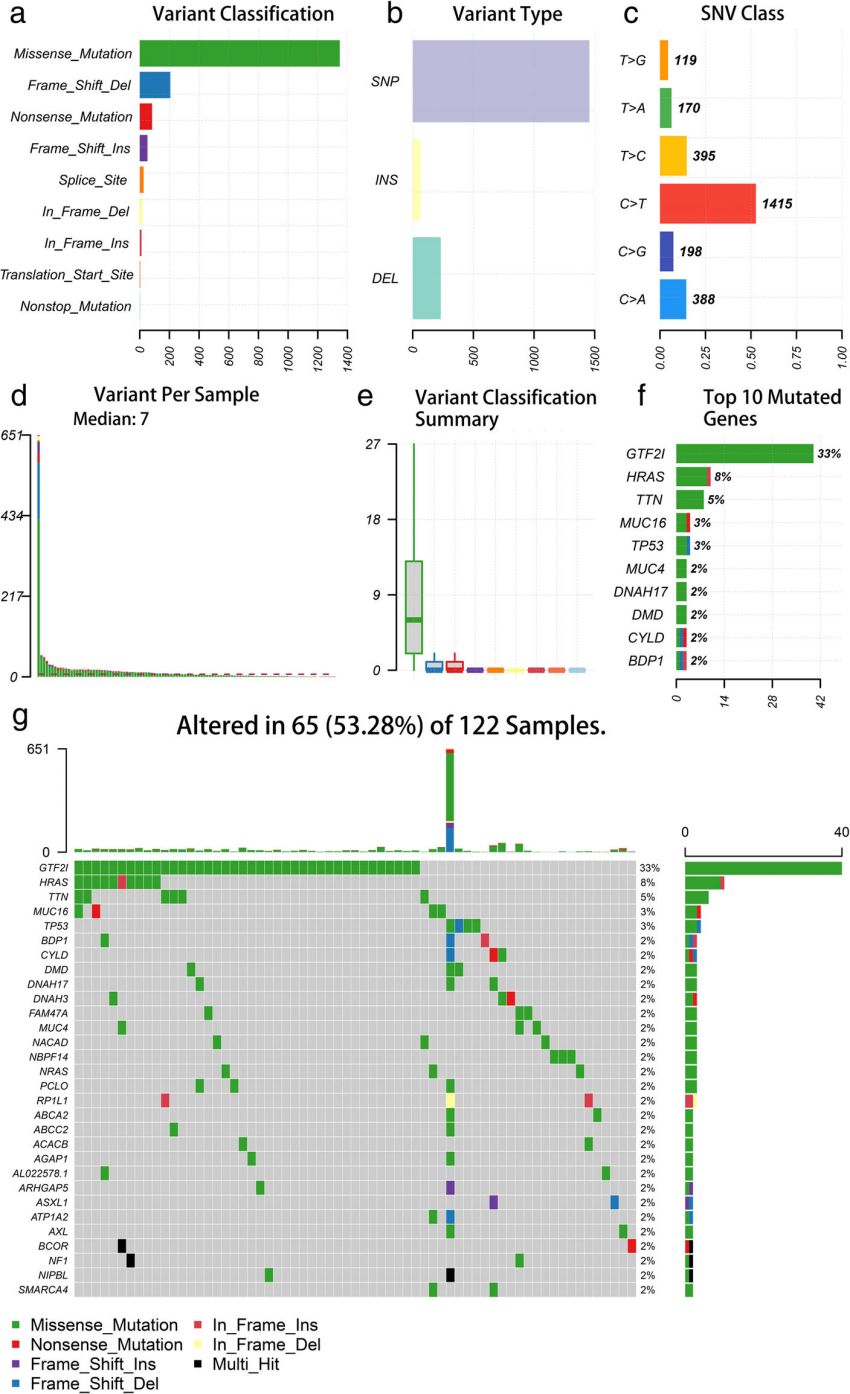
Summary of the genetic mutation information of thymic epithelial tumor (TET) patients. (a, b) Missense mutation accounts for the most fractions in the variant classification and SNP was the most common type of mutation; (c) the SNV class of TETs; (d, e) tumor mutation burden in specific samples; (f) the top 10 mutated genes in TETs; (g) water plot of mutation profiles in each TET sample. SNP, single nucleotide polymorphism; SNV, single nucleotide variants

We obtained the somatic mutation profiles of 122 TETs samples from the TCGA database to further study the inherent factors related to TETs mutagenesis. Based on the data with VCF format, the landscape of mutation information was visualized via the “maftools” R package. Missense mutation was the most common mutation type, followed by frameshift and nonsense mutations (Figure 1(a)). In all variant types, single nucleotide polymorphisms (SNPs) represented a larger fraction than insertions or deletions (Figure 1(b)). In TETs, the C > T transition was a prevailing single nucleotide variant (SNV) (Figure 1(c)). Further, the number of altered bases in each sample was counted, and the results are shown in the box plot (Figure 1(d)). Figure 1(e) also presents the mutation type with color marks for TETs. GTF2I (33%), HRAS (8%), TTN (5%), TP53 (3%), and MUC16 (3%) were ranked among the top five mutated genes in the TETs samples (Figure 1(f)). The waterfall plot presents gene mutation data for each sample, where the mutation type is denoted by various colors with annotations (Figure 1(g)).

### Relationship between TMB with clinicopathological factors and survival

**FIGURE 2:**
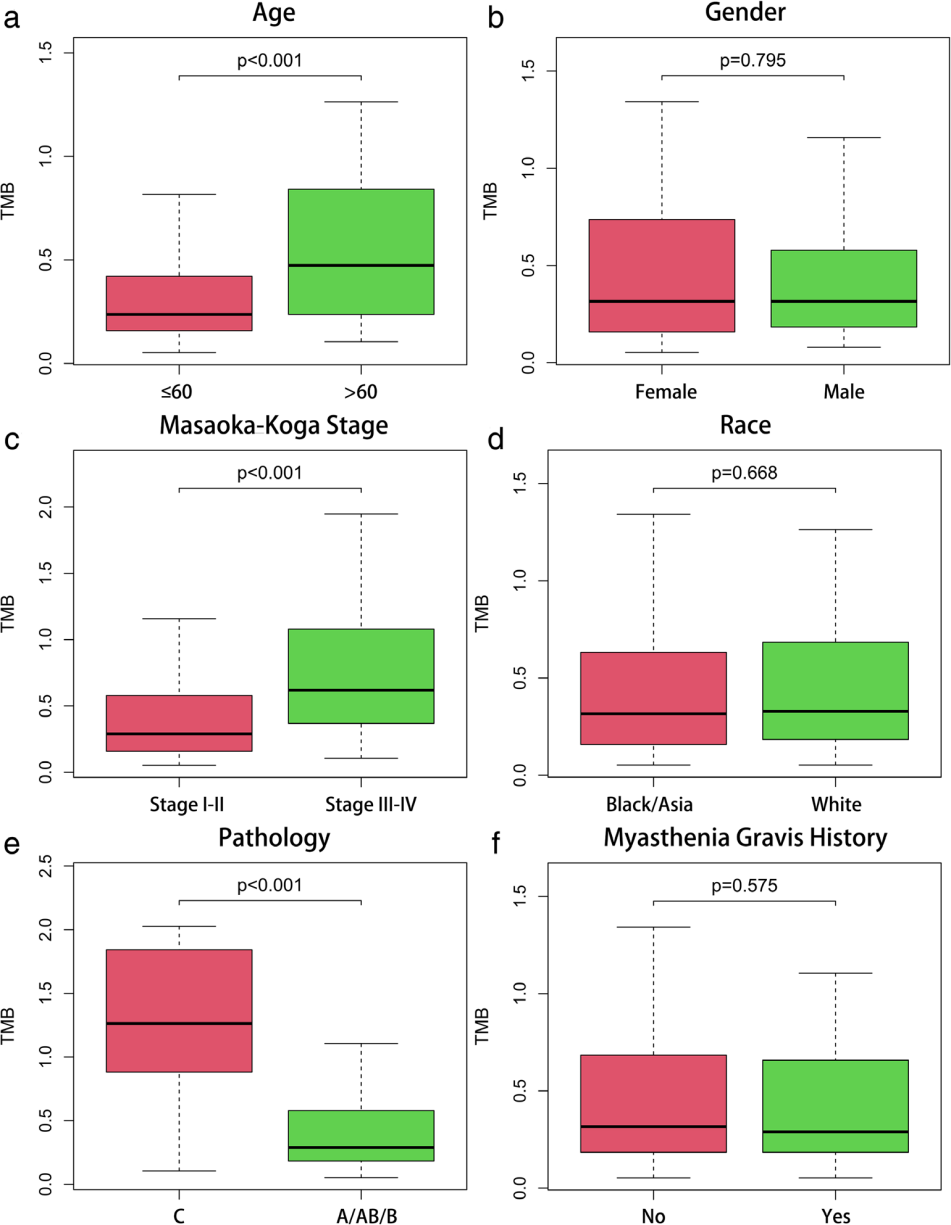
The associations of tumor mutation burden (TMB) in thymic epithelial tumors (TETs) with different clinicopathological characteristics. (a, c, e) Higher TMB level was significantly associated with the older age, later stage and more advanced pathological type. (b, d, f) No significant difference was observed with gender, race, and MG history

**FIGURE 3:**
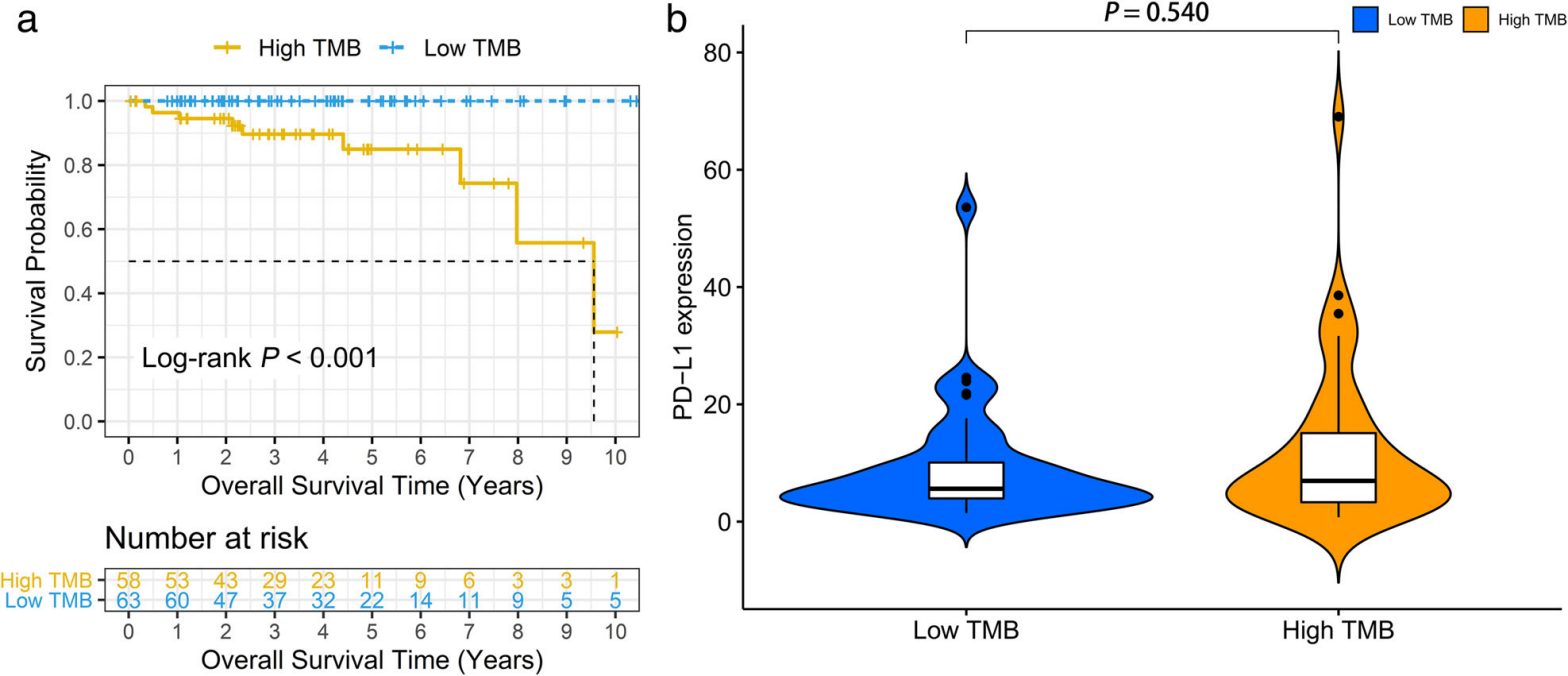
Prognosis of tumor mutation burden (TMB) and associations with PD‐L1 expression. (a) Overall survival of patients with thymic epithelial tumors (TETs) in the high‐TMB group correlated with significantly poor survival outcomes compared with the low‐TMB group (log‐rank *p* < 0.001). (b) No significant difference of PD‐L1 expression level was observed between the high‐ and low‐TMB groups in TETs

The authors calculated the number of mutation events per million bases on tumor samples of 122 patients with TETs as TMB. Based on the median value of TMB, patients were further assigned to the high TMB and low TMB groups. Furthermore, we downloaded the pathological and clinical profiles of all patients and merged the two parts of the data. Prognosis analysis was performed on 121 patients with full survival information. Table 1 shows the clinicopathological characteristics of the two groups. The median age of the patients was 61 years, including 64 men and 68 women. The pathological type of the 111 patients was thymoma, and 11 patients had TC. Subsequently, information on patients' clinicopathological variables was extracted to investigate whether they were correlated with TMB. It was found that a higher TMB was significantly associated with later Masaoka Koga stage, more advanced pathological type, and older age (Figure 2(a), (c), (e)). However, TMB was not correlated with gender, race, or MG history (Figure 2(b), (d), (f)). According to the median value of TMB (0.34), the whole group was divided into high and low TMB groups, and there was no significant difference in the expression of PD‐L1 between the two groups (Figure 3(a), *p* = 0.540). Figure 3(b) shows that the long‐term prognosis of high‐TMB patients was significantly worse than that of the low‐TMB group (10‐year OS: high‐TMB group vs. low‐TMB group: 100% vs. 27.9%, log‐rank *p* < 0.001).

**TABLE 1:** Patient and tumor characteristics in the low‐ and high‐TMB groups

Variables	Low TMB/*N* = 63 (100%)	High TMB/*N* = 59 (100%)	*p*‐value
Gender			0.730
Female	34 (54.0)	30 (50.2)	
Male	29 (46.0)	29 (49.2)	
Age			< 0.001
Mean (±SD)	54.03 (±13.37)	62.90 (±11.09)	
BMI			0.554
Mean (±SD)	27.00 (±5.29)	27.76 (±6.86)	
Prior malignancy			0.661
Yes	4 (6.3)	6 (10.2)	
No	59 (93.7)	53 (89.8)	
Race			0.457
White	50 (79.4)	51 (86.4)	
Black/Asian	11 (22.7)	8 (13.6)	
Unknown	2 (4.5)	0 (0)	
Pathology			< 0.001
Type A	2 (3.2)	15 (25.4)	
Type AB	18 (28.6)	20 (33.9)	
Type B	42 (66.7)	14 (23.7)	
Thymic carcinoma	1 (1.6)	10 (16.9)	
M–K stage			0.003
I–II	56 (88.9)	42 (71.2)	
III–IV	5 (47.9)	17 (28.8)	
Unknown	2 (3.2)	0 (0)	
MG history			0.761
Yes	18 (28.6)	15 (25.4)	
No	44 (69.8)	42 (71.2)	
Unknown	1 (1.6)	2 (3.4)	
Radiotherapy			0.282
Yes	22 (34.9)	15 (25.4)	
No	39 (61.9)	39 (66.1)	
Unknown	2 (3.2)	5 (8.5)	

Abbreviations: BMI, body mass index; MG, myasthenia gravis; MK, Masaoka‐Koga.

### Analysis of DEGs and immune infiltration

**FIGURE 4:**
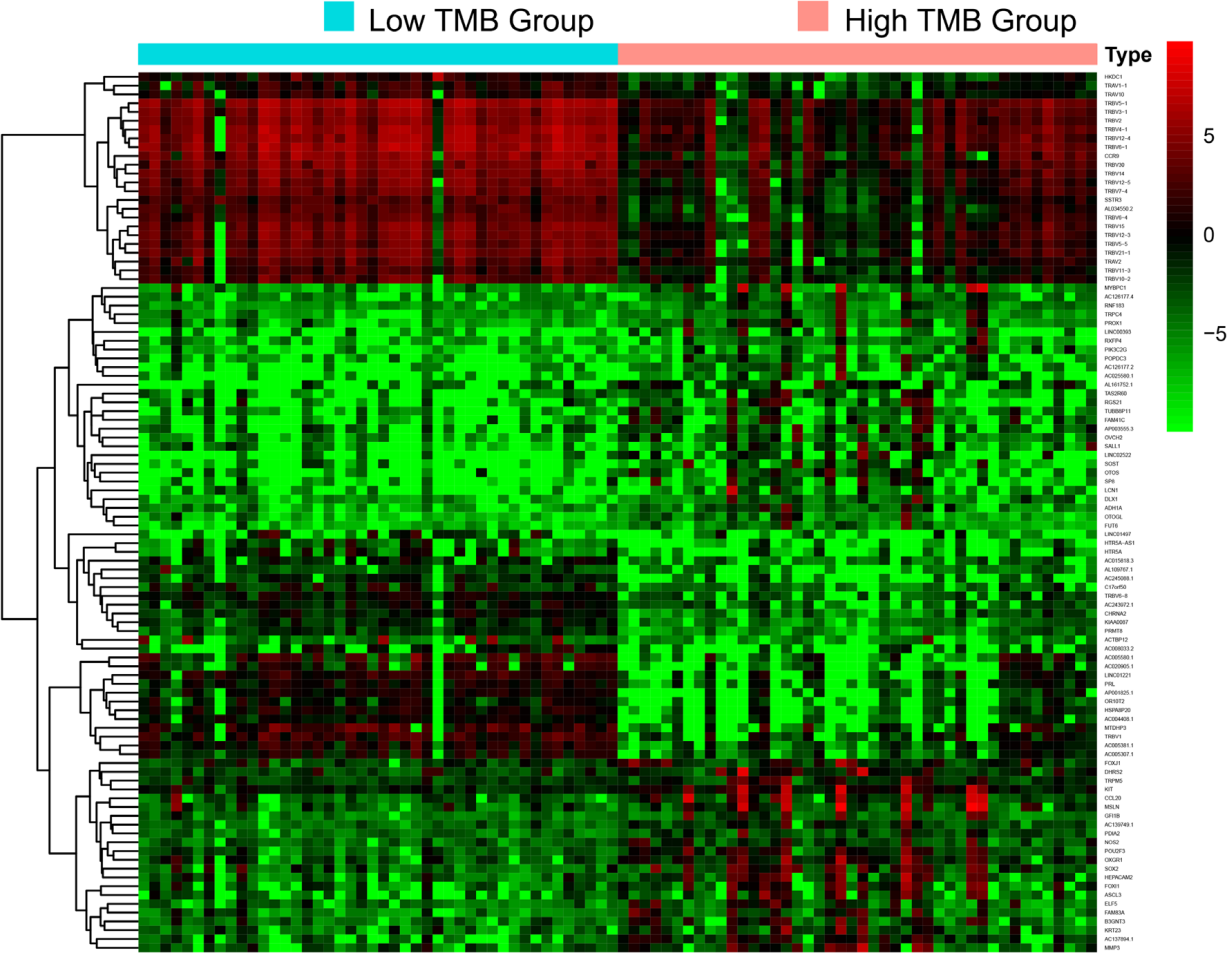
The top 50 differentially expressed genes (DEGs) of the high‐ and low‐ TMB groups are shown in the heatmap plot

**FIGURE 5:**
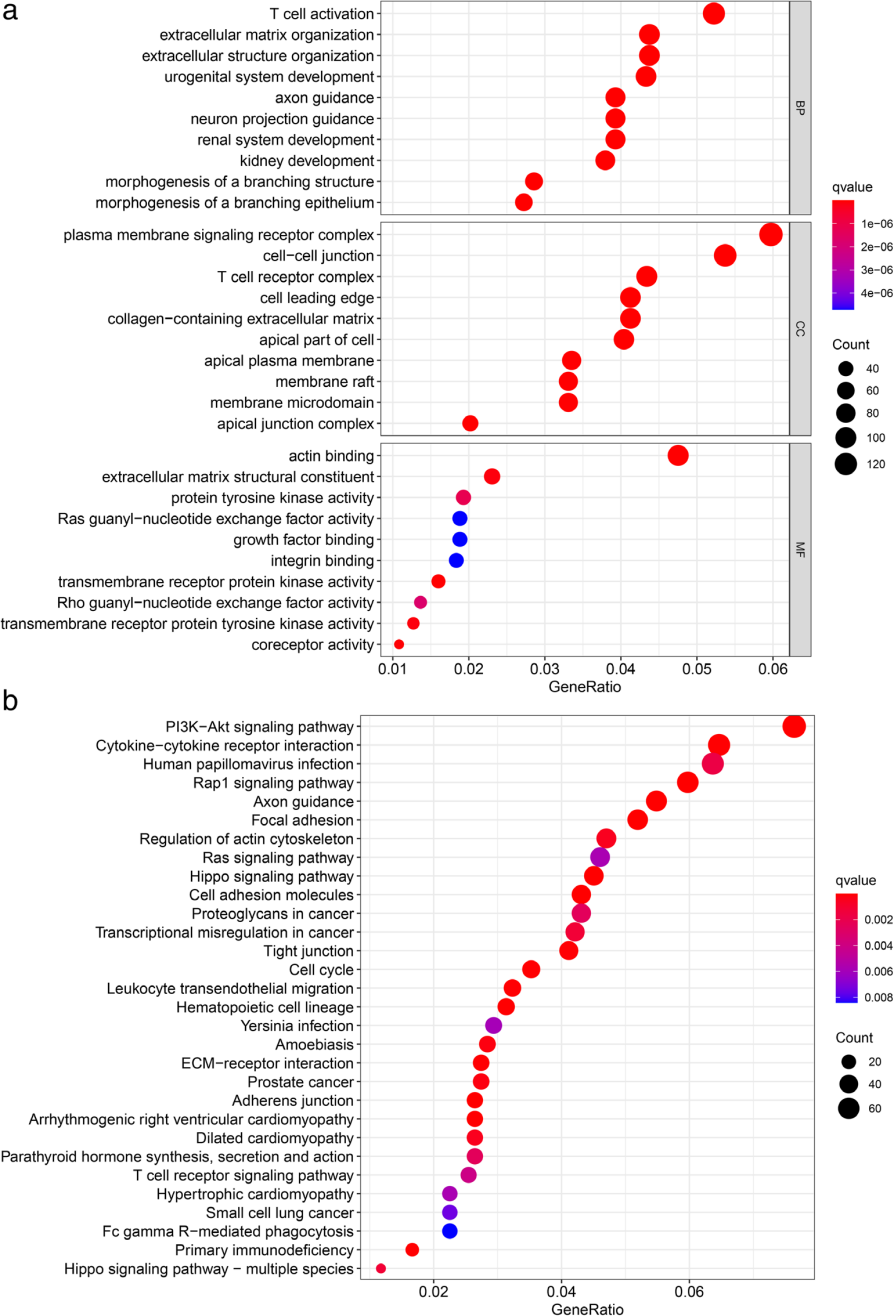
Pathway functional enrichment analysis of differentially expressed genes (DEGs). (a) KEGG enrichment analysis results of DEGs. (b) Gene ontology (GO) enrichment analysis results of DEGs. TMB, tumor mutation burden

**FIGURE 6:**
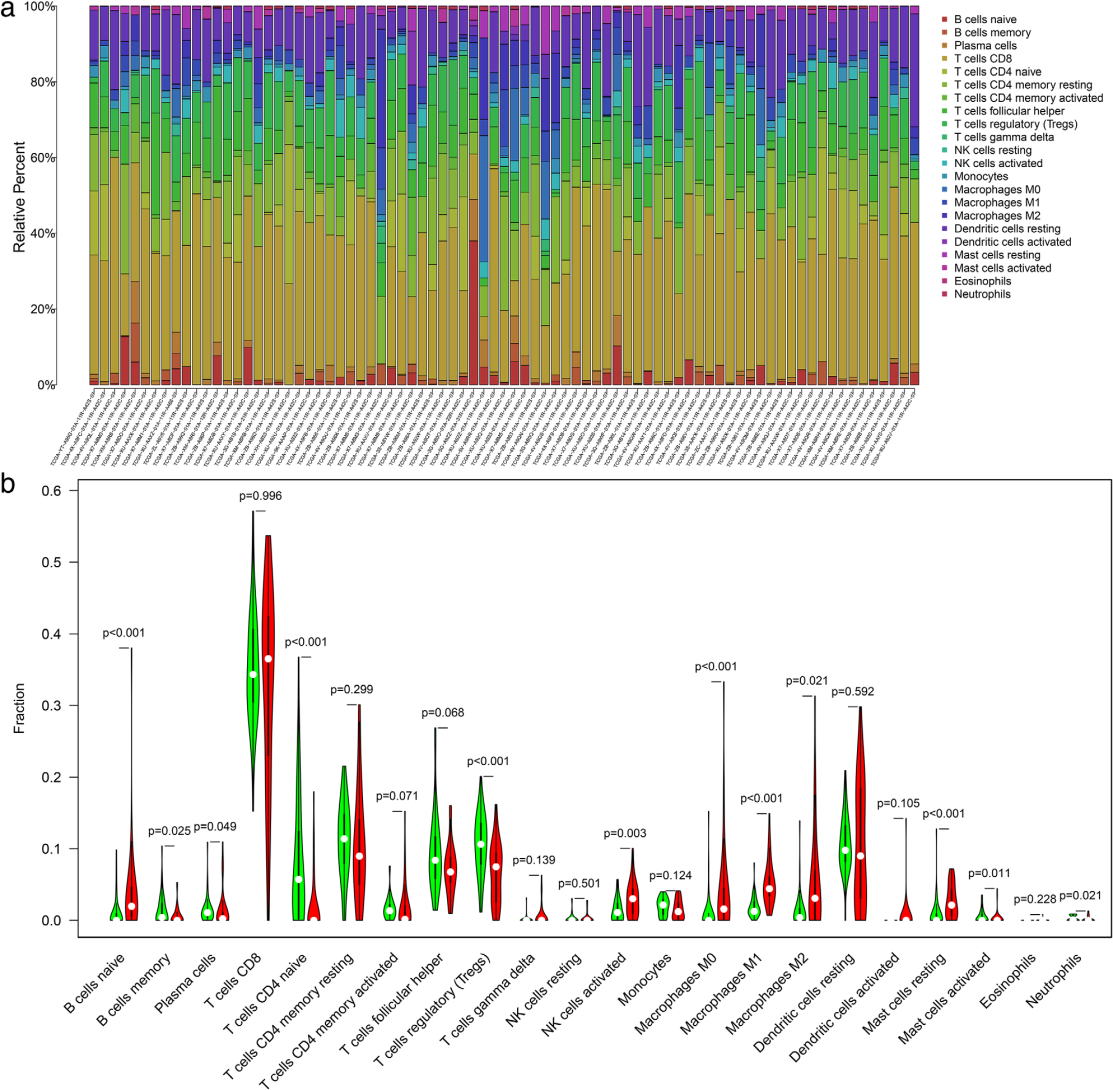
The landscape of immune cells infiltration in thymic epithelial tumors (TETs). (a) The heatmap plot of immune cells infiltration in each sample. (b) The fractions of infiltrated immune cells in high‐ and low–TMB groups. TMB, tumor mutation burden

Differential expression analysis in TETs was performed via the “limma” R package. Eventually, 3795 DEGs with │log2FC│ values >1 and │FDR│ values <0.05, were identified. The hierarchical clustering heatmap presents the top 50 DEGs of the high‐ and low‐TMB groups (Figure 4). Figure 5(a) illustrates the results of the functional analysis, indicating the enrichment of most DEGs in antigen/immunoglobulin receptor binding of molecular function (MF), immunoglobulin component/immune receptor of cellular component (CC), and the immune response of the biological process (BP) by GO analysis. According to KEGG pathway analysis in Figure 5(b), enrichment of DEGs was found in the PI3K‐Akt signaling pathway, cytokine–cytokine receptor interaction, human papillomavirus infection, and Rap1 signaling pathway. We further studied the immune microenvironment in tumor samples. The box plot in Figure 6(a) depicts the specific fractions of the 22 immune cells in each TETs. In addition, there were significant differences in the infiltration levels of plasma cells, activated NK cells, macrophages, resting mast cells, activated mast cells, neutrophils, T cells CD4 naive, regulatory T cells, naive B cells, and B cell memory between the low‐ and high‐TMB groups by Wilcoxon rank‐sum test (Figure 6(b)).

### Identification of irDEGs and PPI analysis

**FIGURE 7:**
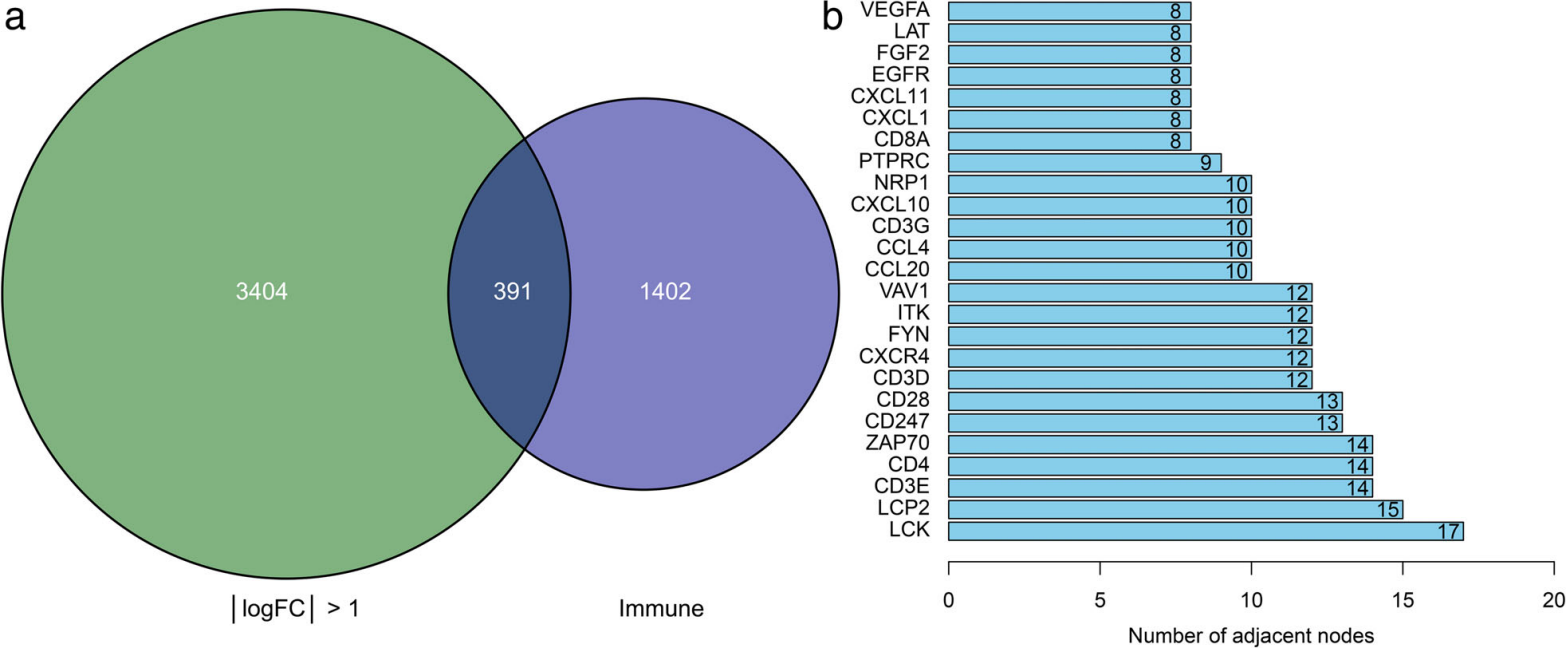
Identification of immune‐related differentially expressed genes (irDEGs) and core genes. (a) Venn plot of DEGs and immune‐related genes, and 391 irDEGs were identified from the two sets. (b) Box plot of top 25 core genes with the highest clustering

**FIGURE 8:**
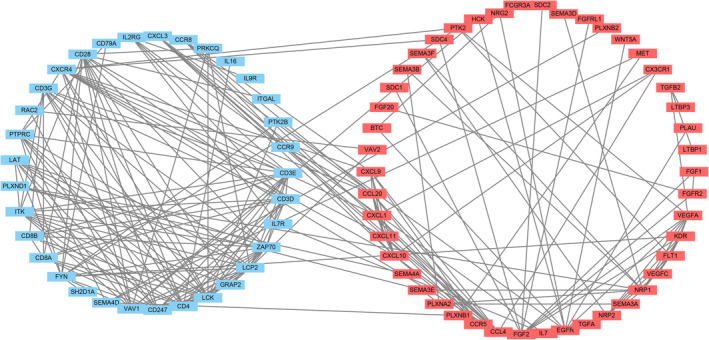
Protein‐to‐protein interaction network analysis of immune‐related differentially expressed genes (irDEGs). The upregulated genes are shown in red and the downregulated in blue

In this study, 1793 immune genes were identified by searching the Immport database. We analyzed the interactions between sets of immune‐related genes and DEGs. In the Venn plot, 391 irDEGs were identified, including 223 downregulated genes and 168 upregulated genes (Figure 7(a)). Among the top 25 core genes with the highest clustering included LCK, LCP2, CD3E, CD4, ZAP70, CD247, and CD28 (Figure 7(b)). To determine the interactions among irDEGs, the STRING online database was used to build the PPI network of the irDEGs, thus identifying major genes participating in tumorigenesis (Figure 8).

### Survival analysis of hub irDEGs signature

**FIGURE 9:**
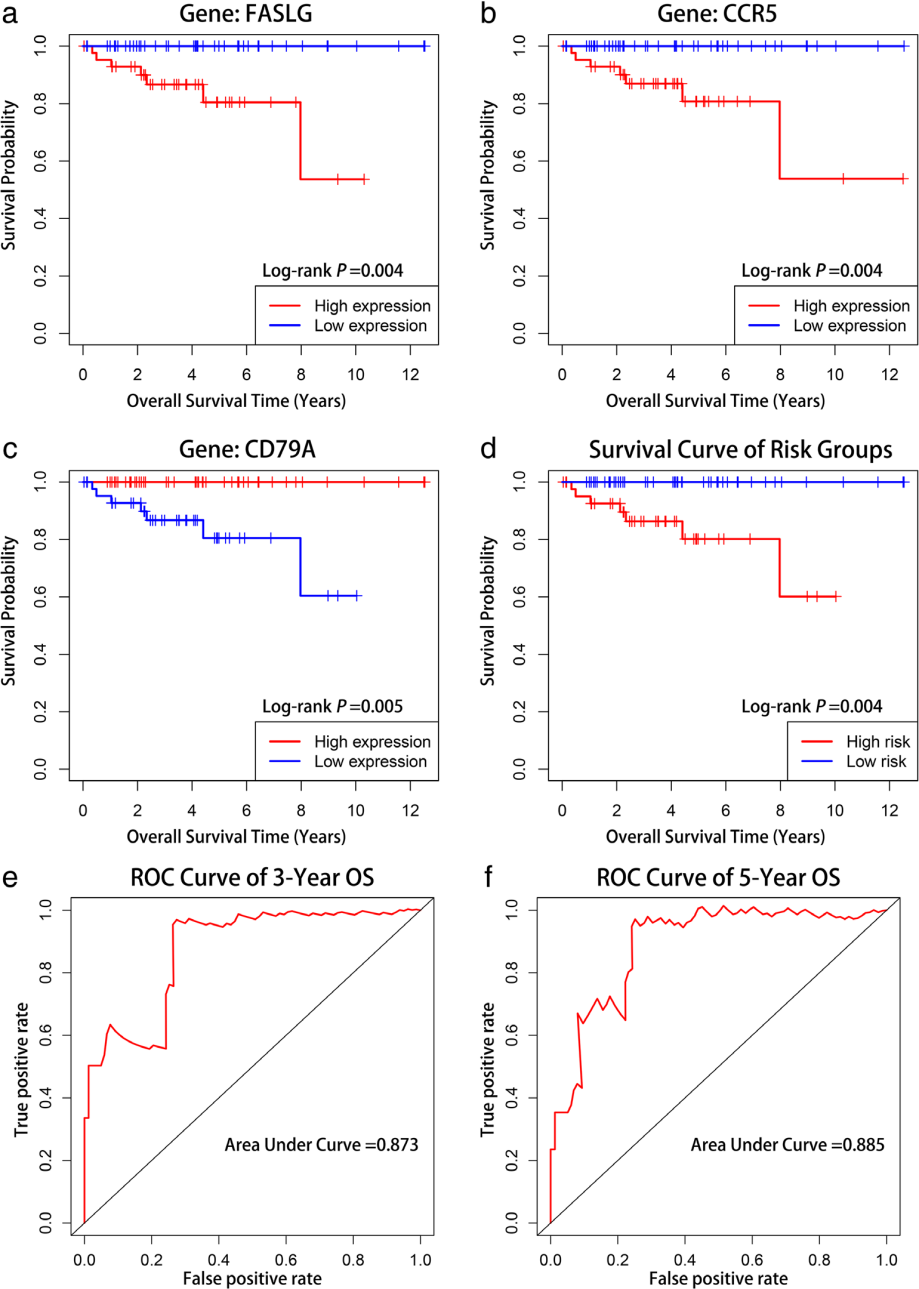
Survival analysis of thymic epithelial tumor (TET) patients with high and low expression of three core genes and survival analysis of gene signature and its predictive ability assessment. Kaplan–Meier curves of (a) FASLG, (b) CCR5, (c) CD79A, and (d) gene signature. The area under the ROC curve of three‐ and five‐year overall survival (OS) for the gene signature were 0.873 and 0.885, respectively (e, f). Receiver operating characteristic curve (ROC)

The Cox proportional hazard model was applied to batch survival analysis, aimed at screening prognostic hub irDEGs with an independent correlation with survival. The genes were CCR5, FASLG, and CD79A. High expression of CCR5 and FASLG was associated with poor prognosis, while high expression of CD79A had a favorable prognosis (Figure 9(a)–(c), Log‐rank *p* < 0.05). We constructed an immune gene scoring signature based on screened hub genes. The median score was used to assign patients to the low‐ and high‐risk groups. Kaplan–Meier analysis revealed a statistically significant difference in prognosis between the two groups (Figure 9(d), log‐rank *p* = 0.004). The prognosis‐related gene signature was constructed and judged using the ROC curve. The area under the curve (AUC) of the gene signature was 0.873 and 0.885 for the three‐ and five‐year OS, respectively, which can be used to evaluate the prognosis of patients (Figure 9(e) and (f)).

## DISCUSSION

The occurrence of tumors is the result of the continuous accumulation of somatic mutations, which mainly include synonymous mutations, insertions, deletions, and base mispairing. In fact, there are essentially nonsynonymous mutations in the development of tumors, especially driver gene mutations that can lead to the occurrence of tumors. When the number of gene mutations accumulates, more new antigens are produced, and there is a greater possibility of being recognized by the immune system.^6^ TMB can indirectly reflect the ability and extent of tumors to produce new antigens. The higher the TMB, the more antigens may be produced, the higher the antigen immunogenicity of the tumor, the stronger the T cell response and anti‐tumor response, and the more suitable for immunotherapy.^7^ In 2014, TMB as a biomarker predictor was found for the first time to predict the efficacy of CTLA‐4 immunotherapy in patients with advanced melanoma.^8^ It was found that melanoma patients with high levels of TMB tended to have higher efficacy in immune checkpoint inhibitor therapy than those with low levels. Other studies have shown that in NSCLC and urothelial carcinoma patients treated with PD‐1/PD‐L1 immunotherapy, higher TMB was significantly correlated with a higher tumor objective response rate (ORR), longer sustained clinical benefit time, and better progression‐free survival (PFS).^9^ A meta‐analysis study found that TMB has a significant predictive effect on the immunotherapy of 27 tumors, and there is a significant correlation between TMB and ORR.^10^ However, there is no research on the effect of TMB on the prognosis of TETs. Therefore, as a promising biomarker predictor, it is necessary to conduct a TMB correlation analysis of TETs in the basic science of gene mutations.

The thymus is an important immune system organ for the development of T cells. Because of the low incidence of TETs, most immunotherapy studies on these diseases are in the exploratory stage. Giaccone et al. used the PD‐1 humanized antibody pembrolizumab to treat 40 patients with recurrent TC.^11^ The phase II clinical trial found that the overall response rate was 22.5%, and the median duration of response was 22.4 months. Similarly, Cho et al. conducted a phase II clinical trial of 33 patients with TETs who received pembrolizumab.^12^ Among them, 26 patients with TC and 7 patients with thymoma had a partial response rate of 21.2% and a stable disease rate of 57.6%. The average PFS time of the two groups was 6.1 months. Both studies have found that the therapeutic effect in patients with high PD‐L1 expression is better than that of patients with low expression. However, no previous studies have evaluated the correlation between the level of TMB and the effect of immunotherapy or PD‐L1 expression.^13^ This study also found that there was no significant correlation between TMB and PD‐L1 levels in TETs, but higher TMB levels were found in thymic cancer and later staged TETs. TMB may be used as another predictor of the effect of immunotherapy to further screen the dominant population of immunotherapy.

Many studies have found that GTF2I is the most common mutation in TETs, especially for the relatively indolent type A and AB thymoma, but is relatively rare in the more aggressive types B and C.^14^ Previous studies have found that patients with GTF2I mutations have a better prognosis, which may be related to the fact that they are common in relatively less aggressive subtypes. In addition, no significant association was found between this gene mutation and autoimmune diseases.^15^ HRAS is a member of the RAS proto‐oncogene family.^16^ However, the prognostic significance of this gene mutation in TETs remains unclear. Previous studies of TP53 and TNN have found that most of them are common in TC.^17^

In this study, GO and KEGG enrichment analyses of DEGs showed that differential genes were functionally enriched in T cell activation, plasma membrane signaling receptor complex, and actin binding, while KEGG analysis showed that these DEGs were highly enriched in the PI3K‐Akt signaling pathway and cytokine‐cytokine receptor interaction pathways. An increasing number of experimental studies on signal pathways have found that they have an important impact on the occurrence and development of tumors and are of great significance to the targeted therapy of tumors. PI3K‐Akt is an important signaling pathway in the human body, which controls many vital cellular biological processes in tumor occurrence and development, including cell proliferation, cell cycle, autophagy, and angiogenesis by phosphorylating downstream proteins step by step.^18^ The PTEN/PI3K/AKT signaling pathway is a classic anti‐apoptosis signal transduction pathway that can regulate many apoptosis‐related proteins or families, such as the apoptosis inhibitor protein, Bcl‐2 family, and cyclin‐dependent protein kinases. The imbalance of this pathway can lead to the occurrence and development of tumors by regulating the cell cycle, inhibiting apoptosis, promoting cell proliferation, and tumor angiogenesis.^19^ At present, research on this tumor‐related pathway is mainly focused on gastric cancer, breast cancer, and so on, but it has not been reported in the literature on TETs.

The FASLG gene, a member of the superfamily of tumor necrosis factor TNF ligands, is a natural ligand of FAS in vivo, which can transmit death signals to the receptor FAS and initiate cell death signal cascades.^20^ The FAS gene plays an important role in apoptosis signal transduction in many cell types. It has been reported that the activity of the FAS/FASL pathway is decreased in a variety of tumor stem cells and is related to poor prognosis and chemotherapy resistance in many tumors.^21^, ^22^ CCR5, a member of the CC chemokine receptor group, is mainly expressed on immature dendritic cells, natural killer cells, monocytes, and resting memory T lymphocytes.^23^ It not only affects the occurrence and development of tumors through inflammation, but also regulates tumor growth and metastasis through interaction with tumor‐related genes. In recent years, an increasing number of studies have shown that in tumor tissues, the high expression level of CCR5 can be used as a biological indicator of poor prognosis of colorectal cancer, prostate cancer, and breast cancer, and has the potential to be introduced to evaluate tumor invasion and metastasis.^24^, ^25^

The tumor microenvironment is composed of malignant transformed cells, immune cells, mesenchymal cells, and extracellular matrix with different functions, and plays an important role in regulating tumor growth, invasion, and metastasis. Tumor‐associated macrophages (TAMs) are derived from peripheral blood mononuclear macrophages and are one of the main inflammatory cells that infiltrate the tumor microenvironment. In this study, we found that the group with a high tumor mutation had higher macrophage infiltration. M1 is considered to have the function of killing bacteria and tumor cells, and can secrete a variety of proinflammatory cytokines. M2 is generally believed to inhibit the immune response and promote angiogenesis and tissue remodeling.^26^ Previous studies have found that there is a higher proportion of CD163+ in TC than in thymoma. Studies have found that TAMs can secrete a large number of factors, such as EGF and TGF‐B1, that stimulate cell proliferation and survival.^27^, ^28^ In vivo experimental studies have shown that TAMs are necessary for the growth of different types of tumors.^28^ In addition, studies on the relationship between TAMs and tumor cell invasiveness show that TAMs upregulate the expression of proteolytic enzymes, plasmin, MMPs, uPA, and their receptors, degrade the extracellular matrix, destroy the basement membrane, and promote tumor cells to infiltrate into the surrounding tissue.^29^ At the same time, some studies have shown that activating the PI3K/mTOR signal transduction pathway can promote M2 polarization of macrophages, while inhibition of this pathway can lead to M1 polarization.^30^, ^31^ This study also found that differentially expressed genes were enriched in the PI3K/mTOR signaling pathway. Therefore, the correlation between TAMs and TMB and long‐term prognosis can be further studied in the future.

However, this study has some limitations. First, some of the data from the public database are unable to perform a comprehensive analysis of more variables and research endpoints. Due to the low incidence of TETs, the number of cases included in this study was relatively limited. This study failed to conduct basic experiments to analyze TMB and verify the importance of gene signatures in the prognosis of TETs.

This study systematically analyzed the significance of the tumor mutation load in TETs. Patients with high TMB had a significantly poor prognosis. It was found that there were significantly higher TMB in older patients, later stages, and TC. In this study, cluster analysis of differentially expressed genes revealed important related pathways. There are some differences in immune cell infiltration between high and low TMB. At the same time, this study found that the immune‐related gene signature can effectively evaluate the long‐term prognosis of patients with TETs. The authors also hope that there will be more basic experimental research on the significance of TMB in TETs in the future.

## CONFLICT OF INTEREST

The authors declare that the research was conducted in the absence of any commercial or financial relationships that could be construed as a potential conflict of interest.
